# Efficacy of single low-dose dexamethasone with NEPA for the 168 h prevention of highly or moderately emetogenic chemotherapy

**DOI:** 10.3389/fphar.2025.1622789

**Published:** 2025-09-29

**Authors:** Yuting He, Fanzhuoran Lou, XinTian Huang, Yiqin Zhang, Qunbo Lin, Weijuan Tan, Quan Chen, Xiurong Ren, Huibo Shi, Li Xiao

**Affiliations:** ^1^ Department of Oncology, Zhongshan Hospital of Xiamen University, School of Medicine, Xiamen University, Xiamen, China; ^2^ Institute of Organ Transplantation, Tongji Hospital, Tongji Medical College, Huazhong University of Science and Technology, Wuhan, China; ^3^ Key Laboratory of Organ Transplantation, Ministry of Education, Chinese Academy of Medical Sciences, Wuhan, China

**Keywords:** low-dose, dexamethasone, NEPA, chemotherapy, nausea, vomiting

## Abstract

**Background:**

Dexamethasone (DEX) can cause various side effects, particularly when used over several consecutive days to prevent chemotherapy-induced nausea and vomiting (CINV). Efforts to minimize the dose and frequency of DEX present challenges in managing CINV.

**Methods:**

This single-center, retrospective study included 100 patients with solid tumors undergoing moderately emetogenic chemotherapy (MEC) or highly emetogenic chemotherapy (HEC). Each patient received a single low-dose DEX (8 mg) and NEPA prior to each chemotherapy cycle. The primary efficacy endpoint was the complete response (CR: no emesis, no rescue medication) within 0–168 h post-chemotherapy initiation in cycle 1. The main secondary endpoints were CR during the acute (0–24 h), delayed (24–120 h), and long-delayed (120–168 h) phases.

**Results:**

Between August 2023 and April 2024, a total of 100 patients received 230 chemotherapy cycles, consisting of 67.4% MEC and 32.6% HEC. CR rates rose from 85% in cycle 1–93.1% in cycle 4, with a slight decline during the delayed phase compared to the acute phase. Better outcomes appeared to be associated with fewer risk factors. Treatment was well-tolerated, with only Grade 1 or 2 adverse events reported; constipation and hyperglycemia were the most common. Regression analysis indicated a significant association between diabetes and CR rates (OR 0.09, 95% CI 0.02–0.40, p = 0.002).

**Conclusion:**

Single low-dose DEX (8 mg) with NEPA safely prevents CINV in high-risk patients during MEC/HEC cycles, offering an alternative to minimize the dose and frequency of DEX.

## 1 Introduction

Chemotherapy-induced nausea and vomiting (CINV), a common and distressing side effect of cancer treatment, greatly affects quality of life (QoL) and treatment compliance (Matzka et al., 2018; Vardy et al., 2022). Emetogenic risk varies by antineoplastic agents, categorized as highly, moderately, low or minimally emetogenic chemotherapy (Warr, 2014; Dranitsaris et al., 2016; Lorusso, 2016; Zhao et al., 2023). A triple combination of an Neurokinin-1 Receptor Antagonist (NK-1 RA), a 5-hydroxytryptamine-3 (5-HT3) RA, and multiple-day dexamethasone (DEX), with or without olanzapine, is recommended by evidence-based guidelines for the prevention of acute and delayed CINV caused by moderately emetogenic chemotherapy (MEC) or highly emetogenic chemotherapy (HEC). Among them, the prophylactic dose of DEX is 12 mg or 20 mg on the first day, followed by 8 mg orally/intravenously for 2–3 days after chemotherapy (Kennedy et al., 2024; NCCN, 2024).

The current guideline suggests a multi-day cumulative dose of 40 mg of dexamethasone to prevent CINV caused by MEC/HEC, but it has certain limitations: 1) DEX-associated adverse effects (metabolic disturbances, infections, osteoporosis, etc.) (Suh et al., 2021; Koshi et al., 2022; Vardy et al., 2022) Currently, widely used immunotherapy aims to restrict the glucocorticoid dose within its therapeutic window to ensure a curative effect. The marketing of new antiemetic drugs allows for the reduction or elimination of dexamethasone in CINV management programs. A randomized trial demonstrated noninferiority of palonosetron plus day-1 intravenous DEX versus prolonged DEX in MEC, with comparable complete response rates (CRR) (Aapro et al., 2010). This aligns with an individual patient data meta-analysis showing maintained antiemetic efficacy with DEX-sparing regimens for MEC/anthracycline-cyclophosphamide (AC) chemotherapy (Okada et al., 2019). Similar findings were reported for HEC when combining NK-1 RA and 5-HT3 RA (Ito et al., 2018; Celio et al., 2021). Two meta-analyses confirmed preserved CINV protection with reduced DEX in MEC/HEC (Celio et al., 2019; Gu et al., 2019).

NEPA is a fixed-dose antiemetic combination comprising netupitant (300 mg), a selective NK-1 RA, and palonosetron (0.50 mg), a second-generation 5-HT3 RA (Feyer and Jordan, 2011; Rojas and Slusher, 2012), demonstrates superior efficacy in controlling delayed-phase CINV (25–120 h post-chemotherapy) (Rojas and Slusher, 2012; Stathis et al., 2012; Thomas et al., 2014), potentially enabling DEX dose reduction. Key evidence includes the non-inferior efficacy of NEPA plus single-dose DEX (12 mg) in cisplatin-based regimens (Celio et al., 2022) and superiority over palonosetron plus DEX in a phase 3 trial (Aapro et al., 2014). Notably, Agre et al. further demonstrated that a reduced dexamethasone dose of 8 mg combined with NEPA achieved 100% CRR in HEC patients without rescue medication (Agre et al., 2023). NEPA-based regimens thus allow for reduced corticosteroid use while maintaining effective control of chemotherapy-induced nausea and vomiting.

While the guideline-recommended single-day DEX dose is 12 mg in combination with NK-1 RA and 5-HT3 RA, pursuing a minimal effective dose remains important to further reduce steroid-related toxicities—particularly in patients receiving concurrent immunotherapy or those with comorbidities such as diabetes. Although previous studies and meta-analyses have incorporated 8 mg DEX in multi-day regimens, and the study by Agre et al. demonstrated promising efficacy and safety with an 8 or 12 mg dose alongside NEPA, the use of a single 8 mg DEX dose in combination with NEPA has not been specifically reported (Agre et al., 2023). This study therefore explores the efficacy of a single low-dose DEX (8 mg)—the lowest reported single-day dose used in combination with an NK_1_ RA and a 5-HT_3_ RA for CINV prevention—administered alongside NEPA to prevent CINV in patients receiving HEC or those at high risk for CINV undergoing MEC. The analysis further assesses the regimen’s efficacy, tolerability, and adherence during the 168-h period following chemotherapy.

## 2 Methods

### 2.1 Study design

This was a pragmatic, single-center, multicycle, retrospective cohort study conducted at Zhongshan Hospital of Xiamen University after the approval of ethics committees. Data were retrieved from the electronic medical records of this center between August 2023 and April 2024. All procedures involving the enrolled patients were conducted following the principles of the Declaration of Helsinki.

### 2.2 Patients

This study enrolled patients aged 18–75 years with histologically/cytologically confirmed solid malignancies who were scheduled to receive either HEC or MEC with ≥2 predefined risk factors. To refine risk stratification for MEC, we first identified published predictors of CINV and then classified MEC-treated patients as having high CINV risk if they presented with ≥2 of the following predefined factors: (1) female sex; (2) age <50 years; (3) prior CINV history; (4) alcohol consumption <5 times per week; (5) motion sickness; (6) morning sickness during pregnancy; and (7) pretreatment anxiety or high nausea expectancy (Warr, 2014; Dranitsaris et al., 2016; Lorusso, 2016; Zhao et al., 2023). All participants had Eastern Cooperative Oncology Group (ECOG) performance status of 0–1, adequate organ function, and had completed at least one treatment cycle. Key exclusion criteria included recent radiotherapy (≤1 week), cognitive impairment, corticosteroid contraindications, baseline nausea/vomiting (≤24 h pre-treatment), non-chemotherapy-related emesis (e.g., gastrointestinal obstruction), and concurrent use of interacting antiemetic medications.

### 2.3 Interventions

Patients receiving MEC/HEC were administered a single intravenous dose of DEX (8 mg) and oral NEPA. NEPA was given 60 min, and DEX was given 30 min before chemotherapy on day 1. Rescue medications (metoclopramide or olanzapine) were permitted based on predefined criteria: if the patient experienced one or more episodes of vomiting or moderate to severe nausea (defined as a nausea score ≥3 according to the Common Terminology Criteria for Adverse Events version 5.0 grading scale) that was subjectively distressing and prompted a request for intervention, within 7 days after chemotherapy administration. Each patient was required to complete at least one cycle, with follow-up extending up to a maximum of four cycles.

### 2.4 Study outcomes

The primary endpoint was the CR rate (defined as no emetic and no rescue medication use) during the whole period (0–168 h) after the infusion of chemotherapy in each course of treatment. Secondary endpoints were defined as follows during the overall, acute (0–24 h), delayed (24–120 h), and long-delayed (120–168 h) period: CR (not counted the whole period), NSN (defined as grade ≤ 1 nausea), CP (defined as no emetic episodes, no use of rescue medication, and no more than mild nausea), no emesis, no nausea, and no use of rescue medication.

Patients recorded daily nausea/vomiting episodes and rescue medication use in diaries throughout each 7-day cycle, with nausea/vomiting severity graded per Common Terminology Criteria for Adverse Events (CTCAE) version 5.0. To enhance data accuracy and consistency, clinical research staff conducted structured telephone follow-ups within 24–48 h after chemotherapy and again on day 7, using CTCAE v5.0 to verify and grade all patient-reported symptoms. Investigator-prescribed rescue medications (metoclopramide/olanzapine) constituted treatment failure. All adverse events (AEs), including glucose monitoring in diabetic/non-diabetic patients, were systematically assessed and documented according to CTCAE v5.0 criteria. Any inconsistencies or ambiguities in diary entries were clarified during follow-up contacts to ensure reliable and standardized data collection.

### 2.5 Statistical analysis

Descriptive analyses presented continuous variables as median (interquartile range, IQR) or mean ± standard deviation (SD) (Shapiro-Wilk test) and categorical variables as frequencies (%). CR and NSN rates across phases were compared using McNemar’s test. Logistic regression identified risk factors for CR. Univariable analysis (p < 0.05) preceded multivariable modeling (variance inflation factor <5, correlation coefficient <0.8), with bootstrap-corrected area under the curve (AUC) evaluating predictive performance. Analyses used Statistical Package for the Social Sciences (SPSS, version 26.0); frequency bar charts and regression visualizations were generated via GraphPad Prism (version 8). The study adhered to Strengthening the Reporting of Observational Studies in Epidemiology (STROBE) guidelines.

## 3 Results

### 3.1 Patient characteristics

**FIGURE 1:**
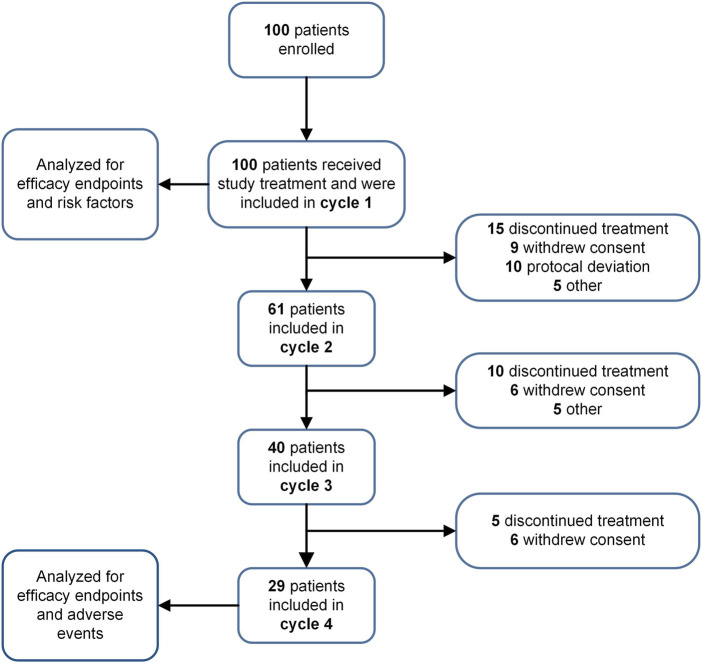
Study flow chart. The progression of patient participation and discontinuation throughout various cycles of a study is depicted.

This study enrolled 100 eligible patients receiving HEC/MEC with ≥2 risk factors constituting the safety population for efficacy and risk factor analysis. A total of 230 evaluable chemotherapy cycles were included, with 29 patients completing all four cycles (Figure 1). The baseline characteristics are summarized in Table 1. The most common cancers were colorectal, lung, and gastric cancers, with other types including esophageal, breast, liver, and ovarian carcinomas. HEC regimens primarily included carboplatin (AUC ≥4; 21%), cisplatin (3%), anthracycline-cyclophosphamide (2%), and DS8201 (5%). MEC regimens primarily included carboplatin (AUC <4; 26%), oxaliplatin (20%), and irinotecan (19%). All patients received a single day of chemotherapy.

**TABLE 1:** Patient’s demographic and clinical characteristics.

Characteristic	Cycle 1 No. (%)	Cycle 2 No. (%)	Cycle 3 No. (%)	Cycle 4 No. (%)
Total number	100	61	40	29
Age, years				
Median (range)/Mean (SD)	60 (48–69)	58 (48–69)	55 (45–71)	55.2 ± 14.8
<60	50 (50)	36 (59)	23 (57.5)	18 (62.1)
≥60	50 (50)	25 (41)	17 (42.5)	11 (37.9)
Sex				
Male	38 (38)	23 (37.7)	14 (35)	10 (34.5)
Female	62 (62)	38 (62.3)	26 (65)	19 (65.5)
BMI (kg/m^2^)				
Mean (SD)	22.2 (3.5)	22.3 (3.8)	22.5 (4.1)	22.1 (4.3)
<22	44 (44)	30 (49.2)	20 (50)	17 (58.6)
≥22	56 (56)	31 (50.8)	20 (50)	12 (41.4)
Cancer type				
Gastric	13 (13)	7 (11.4)	5 (12.5)	5 (17.2)
Colorectal	27 (27)	17 (27.8)	12 (29)	10 (34.4)
Lung	18 (18)	13 (21.3)	5 (12.5)	4 (13.7)
Other	42 (42)	24 (39.3)	18 (45)	10 (34.4)
ECOG performance status				
0	76 (76)	49 (80.3)	36 (90)	28 (96.6)
1	24 (24)	12 (19.7)	4 (10)	1 (3.4)
Alcohol consumption				
Yes	6 (6)	3 (4.9)	3 (7.5)	2 (6.9)
No	94 (94)	58 (95.1)	37 (92.5)	27 (93.1)
Smoking				
Yes	13 (13)	7 (11.5)	4 (10)	4 (13.8)
No	87 (87)	54 (88.5)	36 (90)	25 (86.2)
Tumor stage				
Early	26 (26)	16 (26.2)	8 (20)	7 (24.1)
Metastatic	74 (74)	45 (73.8)	32 (80)	22 (75.9)
Chemotherapy naive				
Yes	35 (35)	0 (0)	0 (0)	0 (0)
No	65 (65)	61 (100)	40 (100)	29 (100)
Chemotherapy (Cycle1)				
MEC (total)	69 (69)	42 (68.9)	26 (65)	18 (62.1)
MEC (2 high-risk factors)	40 (40)	19 (31.1)	9 (22.5)	4 (13.8)
MEC (3–4 high-risk factors)	20 (20)	16 (26.2)	13 (32.5)	9 (30)
MEC (5–6 high-risk factors)	9 (9)	7 (11.5)	4 (10)	5 (17.2)
HEC	31 (30)	19 (31.1)	14 (35)	11 (37.9)
Diabetes mellitus				
Yes	13 (13)	7 (11.5)	6 (15)	4 (13.8)
No	87 (87)	54 (88.5)	34 (85)	25 (86.2)

Data are expressed as the median (inter-quartile range) or number (%).

^a^ It is defined as continuous smoking or drinking for at least 1 month prior to the start of the study.

Abbreviations: DEX, dexamethasone; NEPA, fixed-dose combination of netupitant and palonosetron; BMI, body mass index; ECOG, eastern cooperative oncology group; MEC, moderately emetogenic chemotherapy; HEC, highly emetogenic chemotherapy.

### 3.2 Efficacy

**FIGURE 2:**
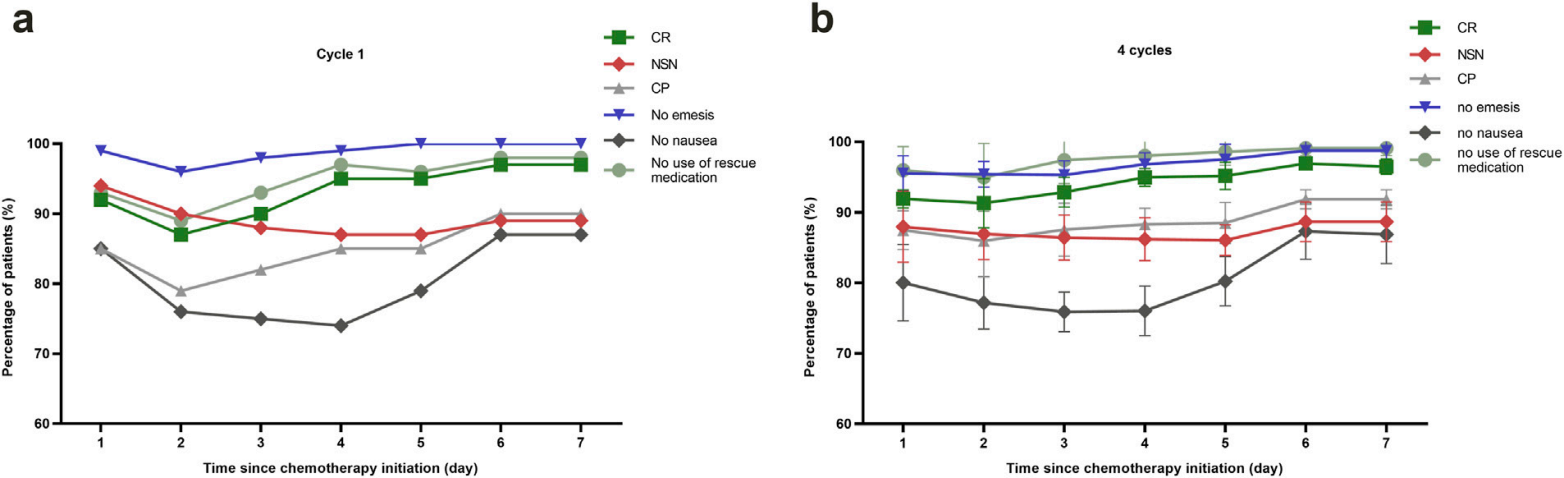
Efficacy assessments of CINV-related outcomes. **(a)** Line graph showing the proportion of patients with different CINV-related outcomes of cycle 1 up to 168 h post-chemotherapy. **(b)** Line graph offering a comparative analysis of patient outcomes related to CINV, tracking changes over the course of four chemotherapy cycles. Abbreviations: CR, complete response; NSN, no significant nausea; CP, complete protection.

Table 2 summarizes the CR and no significant nausea (NSN) rates across acute, delayed, long-delayed, and overall phases. Therapeutic efficacy increased with each successive cycle, as indicated by rising CR and NSN rates. To address potential concerns regarding selection bias from patient dropout, we conducted a Generalized Estimating Equations (GEE) analysis. This analysis confirmed a statistically significant overall effect of treatment cycle on CR rates (Wald χ^2^ = 6.067, p = 0.048) while demonstrating no significant effect of dropout status (Wald χ^2^ = 1.093, p = 0.296), supporting that the efficacy improvement represents a sustained treatment effect rather than artifact from selective attrition. In the first cycle, delayed-phase CINV control was weaker compared to the acute phase by 7%, but this difference decreased in cycles 2 through 4. Figure 2 illustrates endpoint trends over 7 days post-chemotherapy for the first cycle (Figure 2a) and all four cycles (Figure 2b). Both the acute and long-delayed phases consistently showed better results than the delayed phase. McNemar’s test (Supplementary Figure S1) statistically validated this observation for Cycle 1, showing consistent directional trends despite a small sample size.

**TABLE 2:** Analysis of efficacy end-points of different periods in patients receiving a single dose of DEX plus NEPA.

Efficacy endpoint	Overall period (0–168 h)		Acute period (0–24 h)		Delayed period (>24–120 h)		Long-delayed period (120–168 h)	
	*N*	*%*	*N*	*%*	*N*	*%*	*N*	*%*
Cycle 1 (N = 100)								
CR	85	85.0	92	92.0	85	85.0	96	96.0
NSN	87	87.0	94	94.0	86	86.0	89	89.0
Cycle 2 (N = 61)								
CR	54	88.5	46	75.4	45	73.8	48	78.7
NSN	52	85.2	52	85.2	52	85.2	53	86.8
Cycle 3 (N = 40)								
CR	36	90.0	37	92.5	37	92.5	40	100.0
NSN	34	85.0	36	90.0	34	85.0	37	92.5
Cycle 4 (N = 29)								
CR	27	93.1	27	93.1	27	93.1	28	96.6
NSN	23	79.3	23	79.3	23	79.3	25	86.2

Data was expressed as number (%). Abbreviations: DEX, dexamethasone; NEPA, fixed-dose combination of netupitant and palonosetron; CR, complete response; NSN, no significant nausea.

We observed distinct efficacy patterns across all endpoints, stratified by risk (Supplementary Figure S2). Efficacy was highest in MEC patients with 3-4 risk factors (CR 90%, NSN 95%, CP 90%, no nausea 75%, and no emesis 100%), but decreased with 5-6 factors, indicating a non-linear relationship between the number of risk factors and antiemetic effectiveness. In contrast, HEC demonstrated consistent effectiveness, highlighting that responses vary depending on the treatment regimen. These results support the need for personalized antiemetic strategies based on individual risk.

### 3.3 Safety and tolerability

**FIGURE 3:**
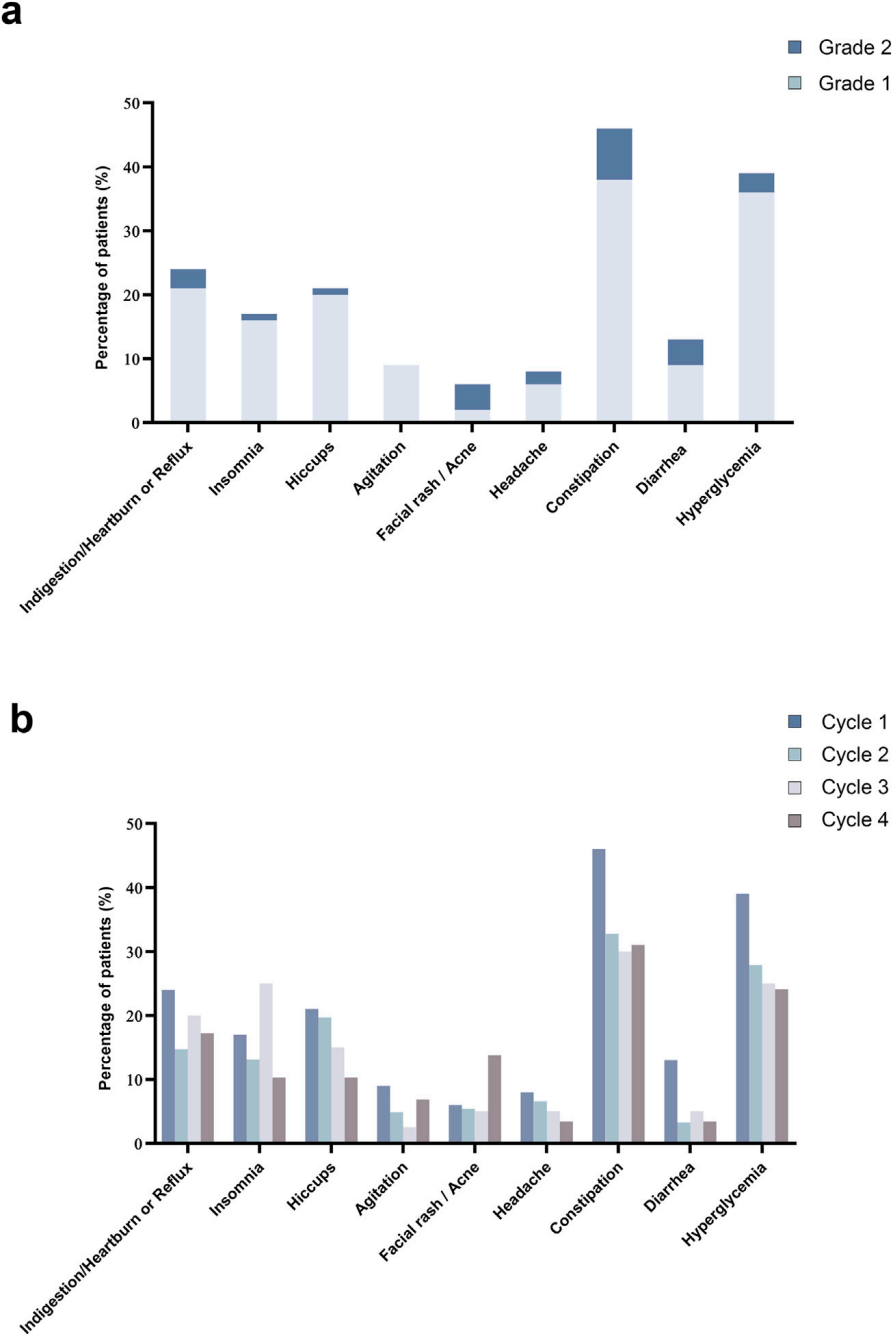
Treatment related adverse events. **(a)** Bar chart displayed the distribution of patients experiencing different grades of TRAEs for cycle 1. **(b)** Bar chart illustrated the percentage of patients experiencing TRAEs, presenting a comparison across chemotherapy cycles 1 through 4.

In this study, all 100 patients completed at least one cycle and documented their experiences in a diary. The majority of patients experienced only mild to moderate (Grade 1 or 2) treatment-related adverse events (TRAEs, Figure 3a); all events were manageable, with no Grade 3–5 TRAEs reported. The incidence of TRAEs remained stable across treatment cycles without evidence of cumulative toxicity. Furthermore, no TRAEs led to treatment discontinuation, and there were no treatment-related deaths. The most common side effects were constipation and hyperglycemia, with no indication of an increase in frequency over multiple cycles (Figure 3b). Hyperglycemia was defined as at least two instances of fasting glucose exceeding 6.1 mmol/L or postprandial (2-h) venous plasma glucose above 7.8 mmol/L within 1 week after chemotherapy across cycles (Alberti and Zimmet, 1998). Among patients with diabetes mellitus, 84.6% (11 out of 13) experienced hyperglycemia, and 23% (3 out of 11) needed to increase their insulin dosage.

### 3.4 Risk factors and predictive model for CR of CINV

We assessed risk factors for CR throughout the entire period using both univariate and multivariate logistic regression (Supplementary Table S1). The following variables were included in the univariate analysis: age, sex, BMI, ECOG performance status, smoking history, alcohol history, presence of distant metastasis, primary tumor site, chemotherapy-naive status, emetogenic risk level of chemotherapy, history of diabetes, prior CINV history, motion sickness, and morning sickness during pregnancy. Variables that reached statistical significance in the univariate analysis, along with those retained after screening for collinearity (VIF <5) and correlation (r < 0.8), were included in the multivariable logistic regression model. These comprised: BMI, ECOG performance status, presence of distant metastasis, emetogenic risk level of chemotherapy, and history of diabetes.

**FIGURE 4:**
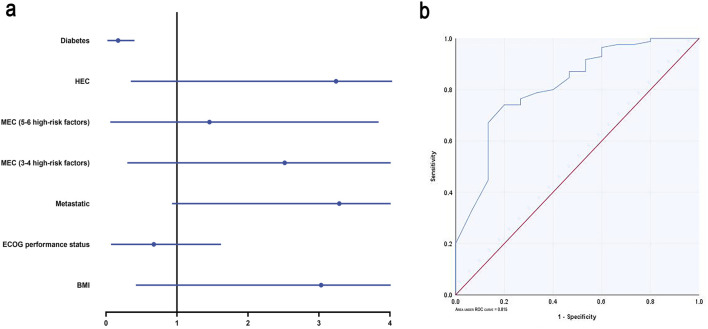
Logistic regression analysis. **(a)** Forest plots of multivariate logistic regression analysis. **(b)** ROC curve according to the multivariate logistic regression. MEC: moderately emetogenic chemotherapy; HEC: highly emetogenic chemotherapy; Abbreviations: CR, complete response; ECOG, Eastern Cooperative Oncology Group; BMI, Body Mass Index; ROC, receiver operating characteristic.

Univariate analysis revealed that ECOG performance status (odds ratio (OR), 0.24; 95% confidence interval (CI), 0.07–0.85; p = 0.027) and diabetes (OR, 0.09; 95% CI, 0.02–0.32; p < 0.001) were significant predictors of CR. Multivariable analysis, accounting for collinearity (VIF <5) and correlation (r <0.8), confirmed that diabetes is the sole independent risk factor (OR 0.09, 95% CI 0.02–0.40, p = 0.002). This corresponds to a CR rate of 46.2% (6/13) in diabetic patients compared to 90.8% (79/87) in non-diabetic patients. Non-diabetic patients exhibited higher CR rates (Figure 4a). The model showed strong discrimination, with an AUC of 0.815 (95% CI 0.70–0.93, p < 0.001). Bootstrap-corrected AUC was 0.817, suggesting minimal overfitting (Figure 4b).

## 4 Discussion

Standard prevention of CINV induced by MEC/HEC typically follows multi-drug regimens recommended by current guidelines. These regimens often include a 5-HT3 RA, an NK-1 RA, and multiday DEX, with or without olanzapine. Guidelines recommended dexamethasone 12 mg once on day 1 followed by 8 mg once on days 2–4. Given the side effects of DEX and the specific dosing needs when combined with certain systemic therapies-immunotherapy etc, we aim to provide a potentially beneficial reduction in DEX dosage for CINV management. Building on findings that NEPA may reduce DEX requirements (Celio et al., 2021; Di Renzo et al., 2020), we investigated the efficacy of a single low dosage of DEX (8 mg) in combination with NEPA for the prevention of CINV induced by HEC or MEC with high risk factors and extended the observation period to 168 h. Our study is the first reported one-day minimum dexamethasone dose administered throughout CINV induced by HEC or MEC with high risk factors.

The half-life of netupitant, a component of NEPA, is 80–96 h, compared to 9–13 h for aprepitant (Navari et al., 2021). Almost all previous antiemetic studies have historically evaluated efficacy only up to 120 h after chemotherapy administration (Kennedy et al., 2024; NCCN, 2024; Natale, 2018). However, CINV symptoms - particularly nausea - frequently persist beyond this timeframe, revealing a critical gap in preventive strategies. Our study spanned a 168-h period, a total of 100 patients receiving MEC or HEC were enrolled. The CR rates during the overall, acute, delayed, and long-delayed periods were 85%, 92%, 85%, and 96% respectively. The line chart analysis revealed that CR and NSN peaked on days 6–7 and maintained a high level during the long-delayed phase. Our finding indicates that future study designs should focus on improving the management of delayed-phase. Delayed nausea and vomiting remain a clinical challenge. Delayed nausea and vomiting primarily occur through the activation of NK1 receptors (Navari et al., 2021; Zhang et al., 2018; Zelek et al., 2021). Di Renzo et al. found that using NEPA alone can achieve an 87% CR rate over the entire 8-day period, with CR values of 88.6% for the acute phase and 98.6% for the delayed phase (Di Renzo et al., 2020). Therefore, a multi-dose regimen of NEPA may be a promising option for strengthening CINV control in the current DEX-sparing approach. Based on the results of our logistic regression analysis, we can identify patients who are more likely to develop CINV and tailor more effective antiemetic treatments for them in future studies.

Our analysis identified patient-related factors as clinically significant contributors to CINV risk in patients receiving MEC (Supplementary Figure S2). Although multivariable logistic regression revealed a non-significant trend toward reduced antiemetic efficacy with increasing risk factors, the consistent pattern suggests that current prophylaxis may be inadequate for MEC patients with high-risk factors, potentially warranting more intensive treatment. Our prediction model, which incorporates ECOG performance status and history of diabetes, demonstrated improved predictability with a bootstrap-corrected AUC of 0.817 (95% CI: 0.68–0.92), indicating clinically useful discrimination for complete response and highlighting the importance of individualized risk assessment. Notably, diabetes mellitus emerged as the sole independent risk factor significantly associated with reduced CR rates (OR 0.09, 95% CI 0.02–0.40, p = 0.002), underscoring its substantial impact on antiemetic outcomes.

These findings support the use of the current regimen as a broadly effective and safe first-line option for most patients. However, we recommend a risk-adapted approach to optimize outcomes: diabetic patients should receive closer glycemic monitoring to manage dexamethasone-induced hyperglycemia—which was manageable in our cohort without treatment discontinuations—while those with poor ECOG status may benefit from intensified support and earlier rescue interventions. Thus, we advocate for widespread use of this regimen with enhanced vigilance in these higher-risk subgroups. These results emphasize the value of personalized risk assessment and tailored management to improve CINV prevention in vulnerable individuals, though validation in larger prospective cohorts is needed to establish precise risk thresholds.

Most previous antiemetic trials focus on a single cycle of treatment, making it difficult to assess the long-term efficacy and safety of these treatments. In our multi-cycle observational study, we observed an increase in the CR rate with each subsequent cycle, approaching nearly 100% by the fourth cycle with the same treatment regimen, without a corresponding increase in TRAEs. Our study assessed the primary treatment-related adverse events (TRAEs) of DEX based on patient diaries. We found that a single low dose of DEX (8 mg) combined with the NEPA regimen was well-tolerated, with no increase in TRAEs across multiple cycles of chemotherapy in both HEC and high-risk patients receiving MEC. Among the participants, 13% had diabetes mellitus, and 39% developed hyperglycemia; however, no additional glycemic control was required during MEC or HEC, further confirming the safety of this single low-dose DEX (8 mg) approach. This simplified regimen may enhance medication adherence and facilitate the clinical nursing process by reducing the occurrence of acute distress caused by CINV.

Our study has several limitations. First, its retrospective design and the absence of a control group may introduce potential biases; therefore, prospective, randomized trials are needed to confirm these efficacy and safety results. Second, future studies in MEC patients should incorporate more individualized risk factors—such as cancer stage, pre-chemotherapy sleep and dietary status, tumor burden, employment, symptom distress, and social functioning—and should also include those with only one high-risk factor to refine risk stratification (Mosa et al., 2020). Third, the CINV prediction model included limited clinical variables and may have omitted other significant predictors. Additionally, as a retrospective analysis, our work focuses primarily on clinical efficacy and safety outcomes. Therefore, further preclinical and translational research is needed to elucidate the exact interactions between neurokinin-1 and 5-HT_3_ receptor inhibition and corticosteroid-mediated antiemetic effects. Despite these limitations, our study demonstrates that a single low-dose dexamethasone (8 mg) regimen combined with NEPA provides effective and safe prophylaxis against CINV in both high-risk MEC and HEC patients over 168 h, offering valuable preliminary insights for optimizing antiemetic strategies.

## 5 Conclusion

This study demonstrates that a single dose of DEX (8 mg) plus NEPA is effective in preventing CINV for a full 168 h in patients receiving HEC, as well as in those with high-risk factors receiving MEC, with sustained efficacy observed across multiple cycles. The regimen demonstrated significant efficacy across all phases, with inferior delayed-phase control. ECOG status and diabetes emerged as significant CR predictors in our model, guiding risk-adapted antiemetic approaches.

## Data Availability

The raw data supporting the conclusions of this article will be made available by the authors, without undue reservation.

## Abbreviations

AC, Anthracycline and cyclophosphamide; AUC, The area under the ROC curve; BMI, Body mass index; CI, Confidence interval; CINV, Chemotherapy-induced nausea and vomiting; CP, Complete protection; CR, Complete response; CTCAE, the Common Terminology Criteria for Adverse Events; DEX, Dexamethasone; ECOG, Eastern Cooperative Oncology Group; GEE, Generalized Estimating Equations; HEC, Highly emetogenic chemotherapy; IPD, Individual Patient Data; MEC, Moderately emetogenic chemotherapy; NK1, Neurokinin 1; NSN, No significant nausea; OR, Odds ratio; QoL, Quality of Life; RA, Receptor antagonists; ROC, Receiver Operating Characteristic; TRAEs, Treatment related adverse events; 5-HT3, 5-hydroxytryptamine-3.
