# Supplementary material for: Efficacy of single low-dose dexamethasone with NEPA for the 168 h prevention of highly or moderately emetogenic chemotherapy
Source: Front Pharmacol. 2025 Sep 29;16:1622789. doi: 10.3389/fphar.2025.1622789 (PMC12515919; doi:10.3389/fphar.2025.1622789)
Supplement: Supplementary file 1 [file Table1.docx]

**Supplementary TABLE 1 Analysis of univariate and multivariate risk factors linked to CR across the entire phase.**

| \| ****Risk factor**** \| \| --- \| |  | ***n*** | **CR** | | **Univariate analysis** | **Multivariate analysis** | |
| --- | --- | --- | --- | --- | --- | --- | --- | --- |
|  |  |  | ***n (%)*** | ***n (%)*** | **OR (95%CI)**  ***P value^α^*** | | **OR (95%CI) *P value*^α^** |
| **Age** | **≥60 vs.<60** | 85 | 40 (40%) | 45 (45%) | 0.44 (0.14-1.41) 0.169 |  | |
| **Sex** | **male vs. female** | 85 | 31 (31%) | 54 (54%) | 0.66 (0.22-1.98) 0.455 |  | |
| **BMI** | **≥22 vs.<22** | 85 | 47 (47%) | 38 (38%) | 0.48 (0.27-2.52) 0.735 | 1.72 (0.42-6.95) 0.450 | |
| **ECOG performance status** | **1 vs. 0** | 85 | 9 (9%) | 76 (76%) | 0.24 (0.07-0.85)  **0.027** | 0.33 (0.07-1.62) 0.171 | |
| **Smoking** | **Yes vs. no** | 85 | 10(10%) | 75 (75%) | 0.87 (0.17-4.43) 0.863 |  | |
| **Alcohol** | **Yes vs. no** | 85 | 3 (3%) | 82 (82%) | 0.24 (0.04-1.56) 0.135 |  | |
| **Metastatic** | **Yes vs. no** | 85 | 65 (65%) | 20 (20%) | 2.17 (0.69-6.83) 0.187 | 3.93 (0.93-16.70) 0.063 | |
| **Site** |  | 85 |  |  |  |  | |
| **Colorectal** | **vs. Gastric** |  | 23 (23%) | 9 (9%) | 1.53 (0.30-7.79) 0.606 |  | |
| **Lung** | **vs. Gastric** |  | 17 (17%) | 9 (9%) | 5.67 (0.51-62.66) 0.157 |  | |
| **other** | **vs. Gastric** |  | 36 (36%) | 9 (9%) | 2.00 (0.42-9.58) 0.386 |  | |
| **Chemotherapy naive** | **Yes vs. no** | 85 | 28 (28%) | 57 (57%) | 0.561 (0.19-1.71) 0.308 |  | |
| **Risk** |  | 85 |  |  |  |  | |
| **MEC (3-4 high-risk factors)** | **vs. MEC (2 high-risk factors)** |  | 18 (18%) | 33 (33%) | 1.91 (0.36-10.17) 0.449 | 2.25 (0.30-16.67) 0.427 | |
| **MEC (5-6 high-risk factors)** | **vs. MEC (2 high-risk factors)** |  | 7 (7%) | 33(33%) | 0.74 (0.13-4.36) 0.742 | 0.47 (0.06-3.84) 0.447 | |
| **HEC** | **vs. MEC (2 high-risk factors)** |  | 27 (27%) | 33 (33%) | 1.43 (0.38-5.41) 0.597 | 1.64 (0.35-7.73) 0.529 | |
| **Diabetes** | **Yes vs. no** | 85 | 6 (6%) | 79 (79%) | 0.09 (0.02-0.32) **0.000** | 0.09 (0.02-0.40) **0.002** | |
| **Prior CINV** | **Yes vs. no** | 85 | 24 (24%) | 76 (76%) | 0.58 (0.18-1.89)  0.363 |  | |
| **Motion sickness** | **Yes vs. no** | 85 | 34 (34%) | 66 (66%) | 0.74 (0.24-2.28)  0.596 |  | |
| **Morning sickness during pregnancy** | **Yes vs. no** | 85 | 25 (25%) | 75 (75%) | 0.90 (0.26-3.14) 0.872 |  | |
| a *P-values* were computed using logistic regression analysis. Abbreviations: CR, complete response; n, number of patients; OR, odds ratio; BMI Body Mass Index; ECOG, Eastern Cooperative Oncology Group, MEC moderately emetogenic chemotherapy; HEC highly emetogenic chemotherapy; CINV Chemotherapy-induced nausea and vomiting. | | | | | | | |
