# Supplementary figures and images for: Efficacy of single low-dose dexamethasone with NEPA for the 168 h prevention of highly or moderately emetogenic chemotherapy
Source: Front Pharmacol. 2025 Sep 29;16:1622789. doi: 10.3389/fphar.2025.1622789 (PMC12515919; doi:10.3389/fphar.2025.1622789)

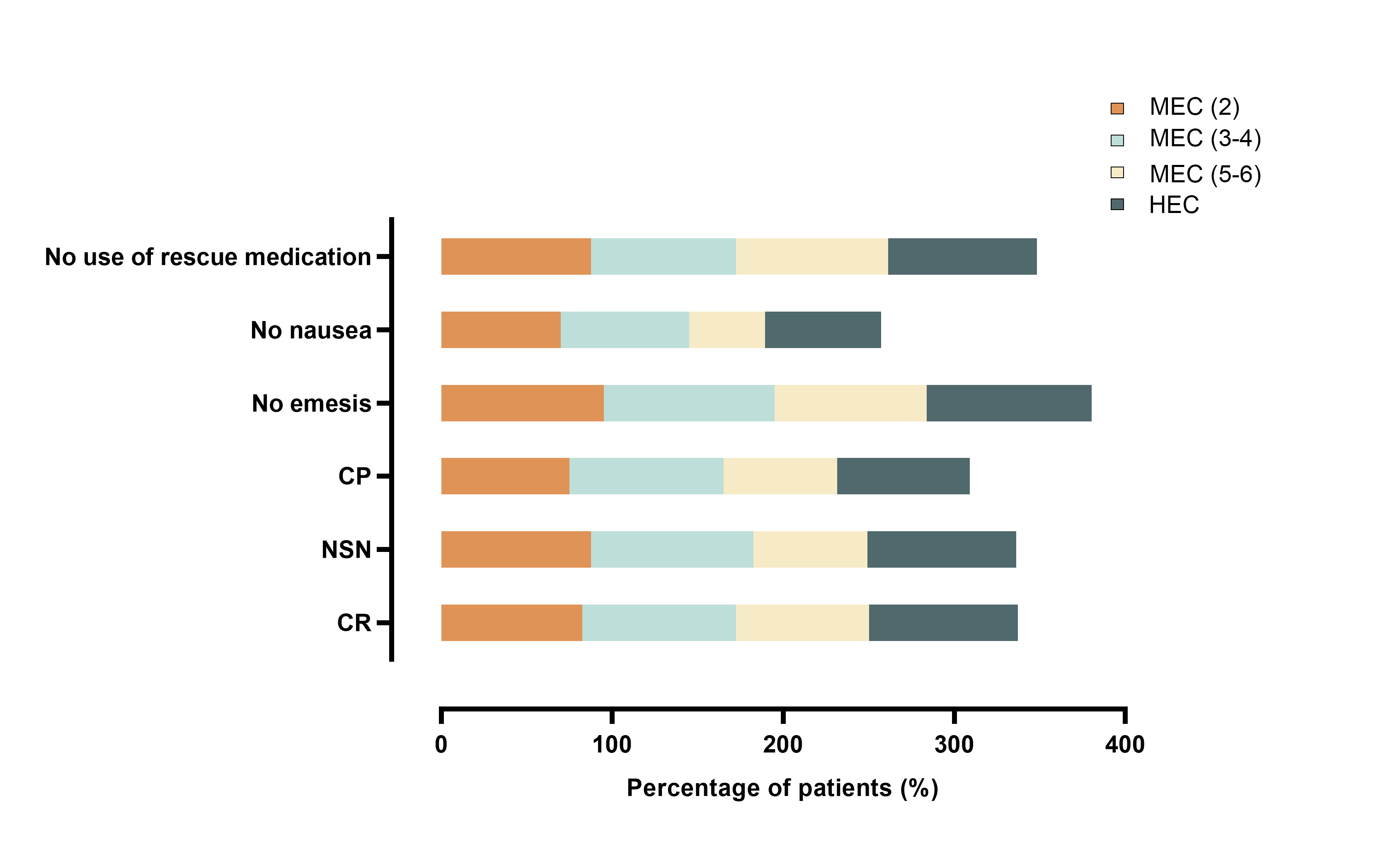

Supplement: Supplementary file 2 [file Image2.tif]

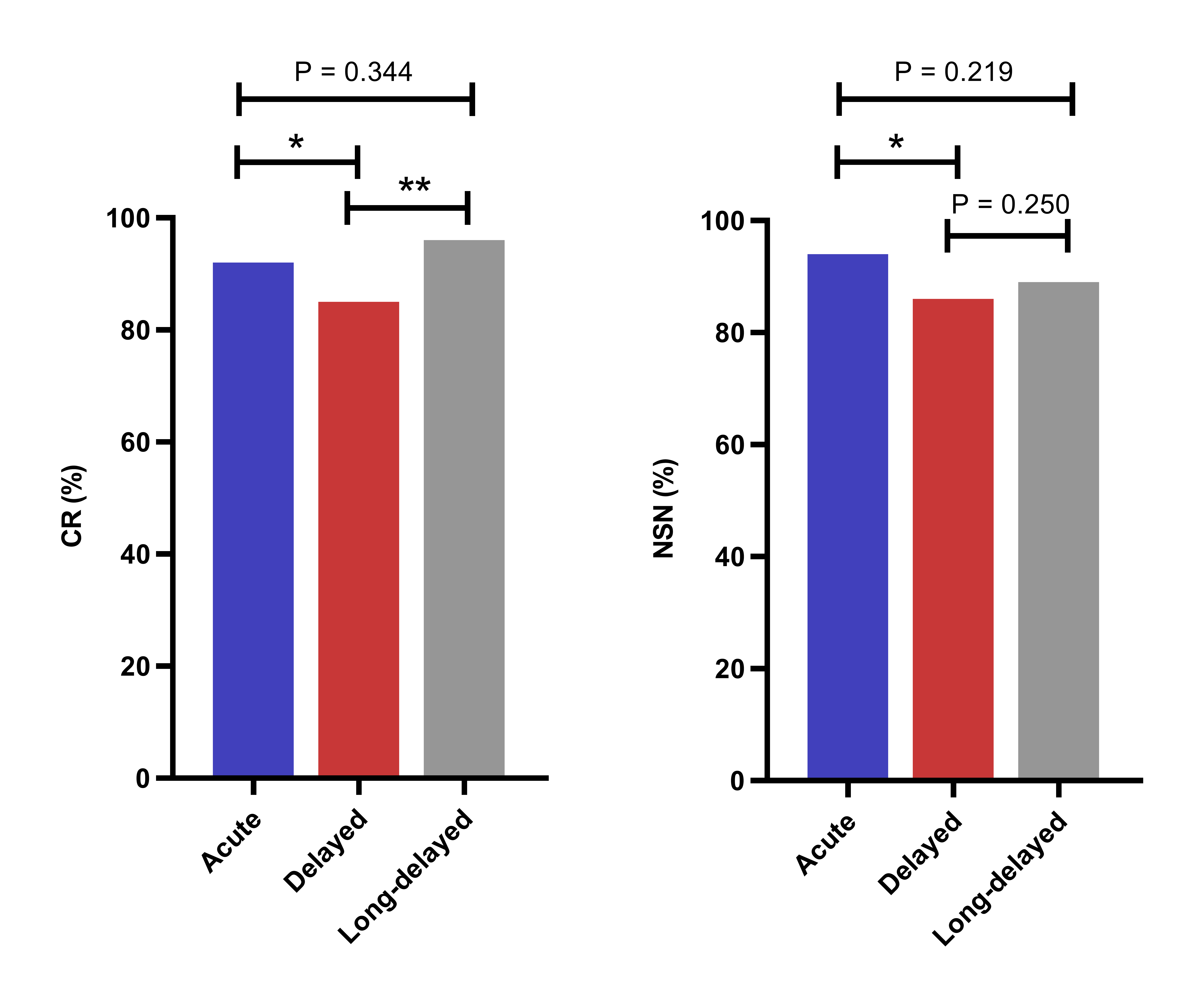

Supplement: Supplementary file 3 [file Image1.tif]
